# Detection of resting-state functional connectivity in the lumbar spinal cord with 3T MRI

**DOI:** 10.1038/s41598-023-45302-0

**Published:** 2023-10-24

**Authors:** Anna Combes, Lipika Narisetti, Anirban Sengupta, Baxter P. Rogers, Grace Sweeney, Logan Prock, Delaney Houston, Colin D. McKnight, John C. Gore, Seth A. Smith, Kristin P. O’Grady

**Affiliations:** 1 Vanderbilt University Institute of Imaging Science, Vanderbilt University Medical Center, 1161 21st Ave S, MCN AA1105, Nashville, TN 37232 USA; 2 Department of Radiology and Radiological Sciences, Vanderbilt University Medical Center, Nashville, TN 37232 USA; 3 Department of Biomedical Engineering, Vanderbilt University, Nashville, TN 37235 USA

**Keywords:** Imaging techniques, Spine regulation and structure, Neurology

## Abstract

Functional MRI (fMRI) of the spinal cord is an expanding area of research with potential to investigate neuronal activity in the central nervous system. We aimed to characterize the functional connectivity features of the human lumbar spinal cord using resting-state fMRI (rs-fMRI) at 3T, using region-based and data-driven analysis approaches. A 3D multi-shot gradient echo resting-state blood oxygenation level dependent-sensitive rs-fMRI protocol was implemented in 26 healthy participants. Average temporal signal-to-noise ratio in the gray matter was 16.35 ± 4.79 after denoising. Evidence of synchronous signal fluctuations in the ventral and dorsal horns with their contralateral counterparts was observed in representative participants using interactive, exploratory seed-based correlations. Group-wise average in-slice Pearson’s correlations were 0.43 ± 0.17 between ventral horns, and 0.48 ± 0.16 between dorsal horns. Group spatial independent component analysis (ICA) was used to identify areas of coherent activity¸ and revealed components within the gray matter corresponding to anatomical regions. Lower-dimensionality ICA revealed bilateral components corresponding to ventral and dorsal networks. Additional separate ICAs were run on two subsets of the participant group, yielding two sets of components that showed visual consistency and moderate spatial overlap. This work shows feasibility of rs-fMRI to probe the functional features and organization of the lumbar spinal cord.

## Introduction

Functional MRI (fMRI) of the spinal cord has been used to detect areas of activation in response to sensory or tactile stimuli, to delineate resting-state functional networks within and between segments, and to investigate changes linked to disease or trauma^1^. While the development of acquisition and analysis methods has focused to date on the cervical cord, the functional organization of the thoracolumbar cord is also of interest.

The lumbar cord is involved in lower limb motor and autonomic functions and has clinical relevance as a location of predilection for disease activity in multiple sclerosis^2^ and a site susceptible to traumatic injury^3^ and disc disease^4^. By lumbar spinal cord, we refer herein to the portion of the cord supplying the lumbar spinal nerve roots responsible for lower limb and some autonomic functions; and specifically the lumbar enlargement, which can be identified on imaging by its larger cross-sectional area and is located adjacent to vertebral levels T11-L1^4^, with some degree of inter-individual anatomical variation^5^. The lumbar enlargement presents some imaging advantages relative to the cervical cord, namely decreased motion and a greater proportion of gray matter (GM), but is also subject to greater magnetic field inhomogeneity due to position within large bones, temporally varying field inhomogeneity due to proximity to the lungs, and the effects of arterial pulsations and cerebrospinal fluid (CSF) flow^6, 7^.

Previous studies in the lumbar cord have relied on a variety of block-design paradigms including thermal^8, 9^ or vibration^10, 11^ stimulation of the lower dermatomes, a lower limb motor task^12^, sexual stimulation^13^, an interoceptive awareness task^14^, and viewing of ‘negative’ visceral stimuli^15^. Only preliminary reports have shown feasibility of measuring functional connectivity in the resting state (rs)^16, 17^. Characterization of the resting-state properties of the spinal cord can inform on its functional features and organization, and has potential to provide novel markers of neurological impairment, with reports of altered cervical intra-segmental connectivity in the presence of lesions^18^ and diffuse tissue damage^19^ in multiple sclerosis, and decreased inter-segmental connectivity in Parkinson’s disease^20^.

Several acquisition strategies have been proposed for acquisition of spinal cord rs-fMRI^21^. Multi-shot gradient echo (GRE) sequences have shown sensitivity to blood oxygenation level dependent (BOLD) effects in the cervical spinal cord^22^ and been validated using hypercapnic and motor paradigms at 3 Tesla (3T)^23^. Based on prior experience optimizing this sequence in the cervical spine, we applied a similar rs-fMRI protocol at 3 Tesla (3T) in healthy volunteers, with the aim of detecting and quantifying rs-functional connectivity in the lumbar cord.

## Methods

### Data acquisition

The study was approved by the Vanderbilt Institutional Review Board Health Sciences Committee, and all research was performed in accordance with relevant guidelines and regulations. Twenty-eight participants with no history of neurological or spinal condition were prospectively enrolled between April 2021 and June 2022, and gave informed consent before study procedures. Two participants were subsequently excluded: one due to faulty physiological data acquisition, one due to an artefact intersecting the cord in the functional run. The remaining dataset consisted of 26 participants (mean age 39.5 ± 12.3 years, 11 males). Scans were performed on a 3T Philips dStream Ingenia MR scanner with 2-channel transmit, and a dStream total spine coil with an integrated 12-channel posterior coil for reception. A triangular foam cushion was placed behind participants’ knees to minimize leg motion, and participants were instructed to remain still. A fingertip pulse oximeter and chest bellows were used for physiological monitoring. After landmarking at the bottom of the ribs, a sagittal T_2_-weighted scan was acquired to help position the field of view. Axial acquisitions were centered at the lumbar enlargement (vertebral levels T11-L1, depending on participant). FMRI parameters were as follows: 3D axial multi-shot gradient echo BOLD-sensitive sequence, volume acquisition time = 2.6 s, TE = 20 ms, *α* = 8°, acquired/reconstructed voxel size 1.1 × 1.1 × 10mm^3^/0.43 × 0.43 × 5mm^3^, 14 slices, field of view = 110 × 101mm^2^, 200 dynamics (~ 9 min), EPI factor = 9, ‘ProSet 1331’ saturation pulse for fat suppression.

### Image processing

**Figure 1:**
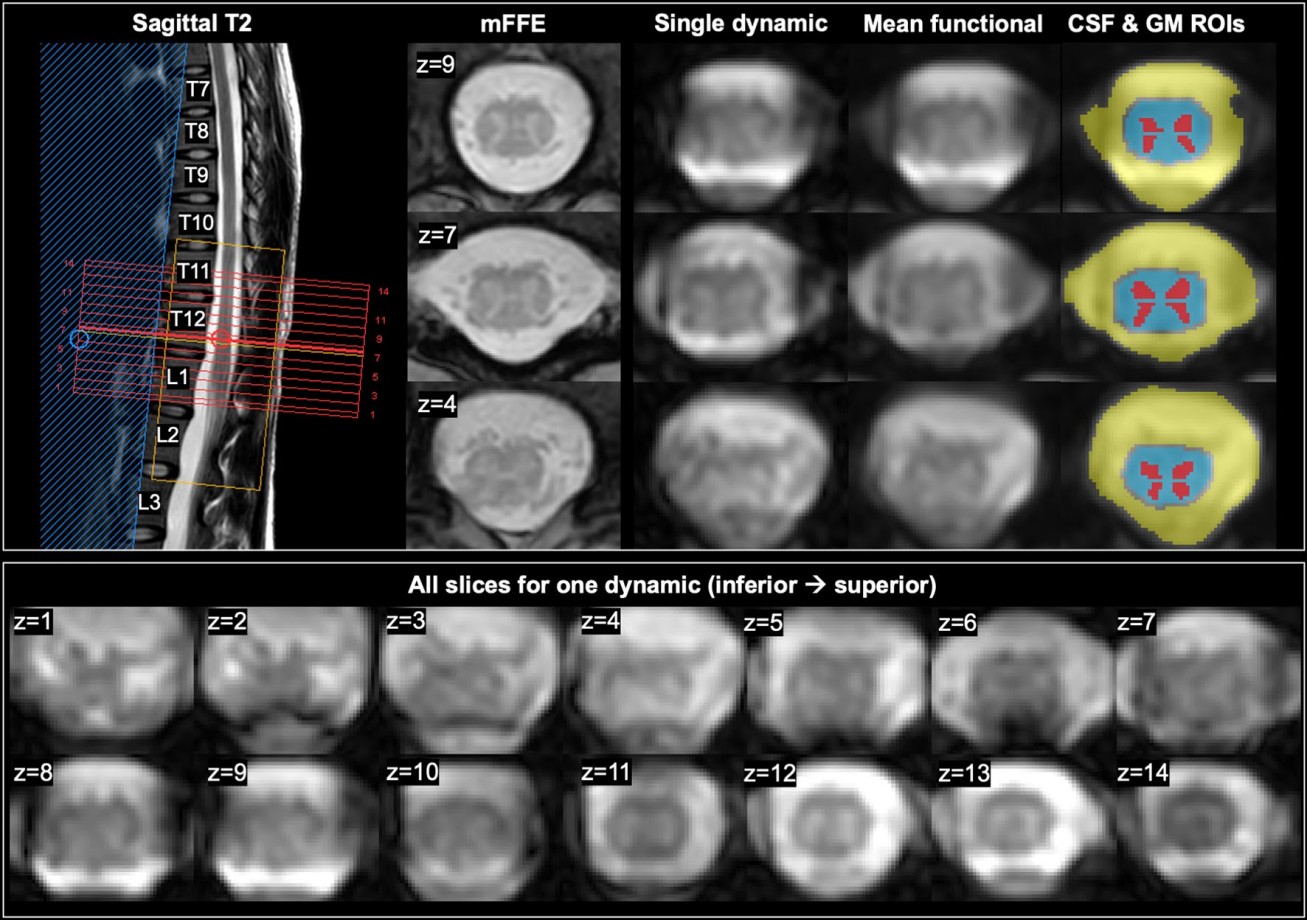
Example acquired images in one participant (26-year-old female). Left: acquired volume (red box) centered at the lumbar enlargement, shown on a sagittal T_2_-weighted scan. A multi-echo gradient echo scan (proton-density/T_2_^*^-weighted axial multi-echo fast field echo (mFFE), acquired voxel size 0.65 × 0.65 × 5mm^3^) is included for visualization of anatomy. Middle to right: a single functional dynamic after motion correction; the mean functional image after denoising; and regions of interest including cerebrospinal fluid (CSF) in yellow (used for nuisance regression), cord in blue, and gray matter (GM) horns in red. Representative slices for one dynamic volume in one participant are shown below, and numbered (z) from inferior (caudal) to superior (rostral).

FMRI processing was performed using FSL v6.0.4^24^, SCT v4.0.2^25^, and in-house code. Slicewise motion correction was performed in SCT with regularization along the rostro-caudal axis and trilinear interpolation; no further spatial smoothing was applied. AFNI-RETROICOR^26^ was used to calculate physiological signal modulations based on recorded cardiac and respiratory traces. CSF and ‘not-spine’ (whole image excluding the spinal canal and cord) masks were created, and principal component analysis was performed slicewise to obtain the five components within each mask explaining the largest amount of variance. The RETROICOR output, CSF and not-spine regressors, and motion correction parameters were then regressed from all time-series in each slice. Temporal band-pass filtering was performed with cut-offs of 0.01 and 0.10 Hz. Cord and GM masks were obtained from the average functional image using *sct_deepseg_sc* and *sct_deepseg_gm* within SCT, and manually corrected when necessary. The GM masks were partitioned into quadrants to define regions of interest (ROIs) in the four horns. This was done by using the center of mass of the mask slicewise to bisect the mask vertically and horizontally, removing three vertical middle slices to exclude the central commissure and one horizontal middle slice to separate anterior and posterior GM (Fig. 1).

### Quality assessment and functional connectivity metrics

Temporal signal-to-noise ratio (TSNR) was computed voxelwise as the mean signal of each time-series over its standard deviation, after motion correction and after nuisance regression separately. Pearson’s correlation coefficients (*r*) were calculated between each ROI-pair within-slice, forming six networks: ventral-ventral, dorsal-dorsal, two ipsilateral ventral-dorsal, and two contralateral ventral-dorsal. TSNR and r coefficients were calculated for each participant as the mean across the central 10 slices. AFNI-InstaCorr^27^ was used to visually explore seed-based correlations in two randomly selected participants.

### Independent component analysis

FMRI data after nuisance regression were cropped to 5 slices centered at the lumbar enlargement (slices 4:8). A participant with good quality data was visually identified and chosen as a target. All scans were registered to this subject’s mean functional image using in-plane nonlinear deformations in SCT and trilinear interpolation. Group spatial independent component analysis (ICA) was performed in Matlab R2018b with the GIFT toolbox v3.0b^28^, using the ‘Infomax’ algorithm, a cord mask, and a preset of 50 components (determined a priori via trial-and-error to maximize the number and appearance of GM components). Components were thresholded at *z* ≥ 4, visually inspected by two independent observers, and identified as belonging to the GM, white matter (WM), or reflecting noise. Empirically-derived criteria for identification of noise components included: smaller spatial extent in the rostro-caudal direction, greater size in-plane, and not following anatomical tissue boundaries. GM components were further classified as ventral, dorsal, and intermediate/central GM based on their anatomical locations and by examining the location of peak *z*-score. As the dimensionality (number of a priori components) of ICA influences output, a second set of ICA parameters was tested with lower dimensionality to maximize the likelihood of observing bilateral components. For this, a GM mask and a preset of 10 components were used on the same data. Finally, to evaluate consistency between the obtained components over the participants, the group was divided into two age- and sex-matched sub-groups (*n* = 13 each, see Supplemental Material), and two separate ICAs were run using the parameters described above. From each ICA output, GM components were identified, and visually matched between Group 1 and Group 2, considering their location in-slice as well as rostro-caudally. Spatial overlap between thresholded (*z* ≥ 4), binarized component maps was measured with Dice coefficients.

## Results

### Quality assessment and functional connectivity metrics

**Figure 2:**
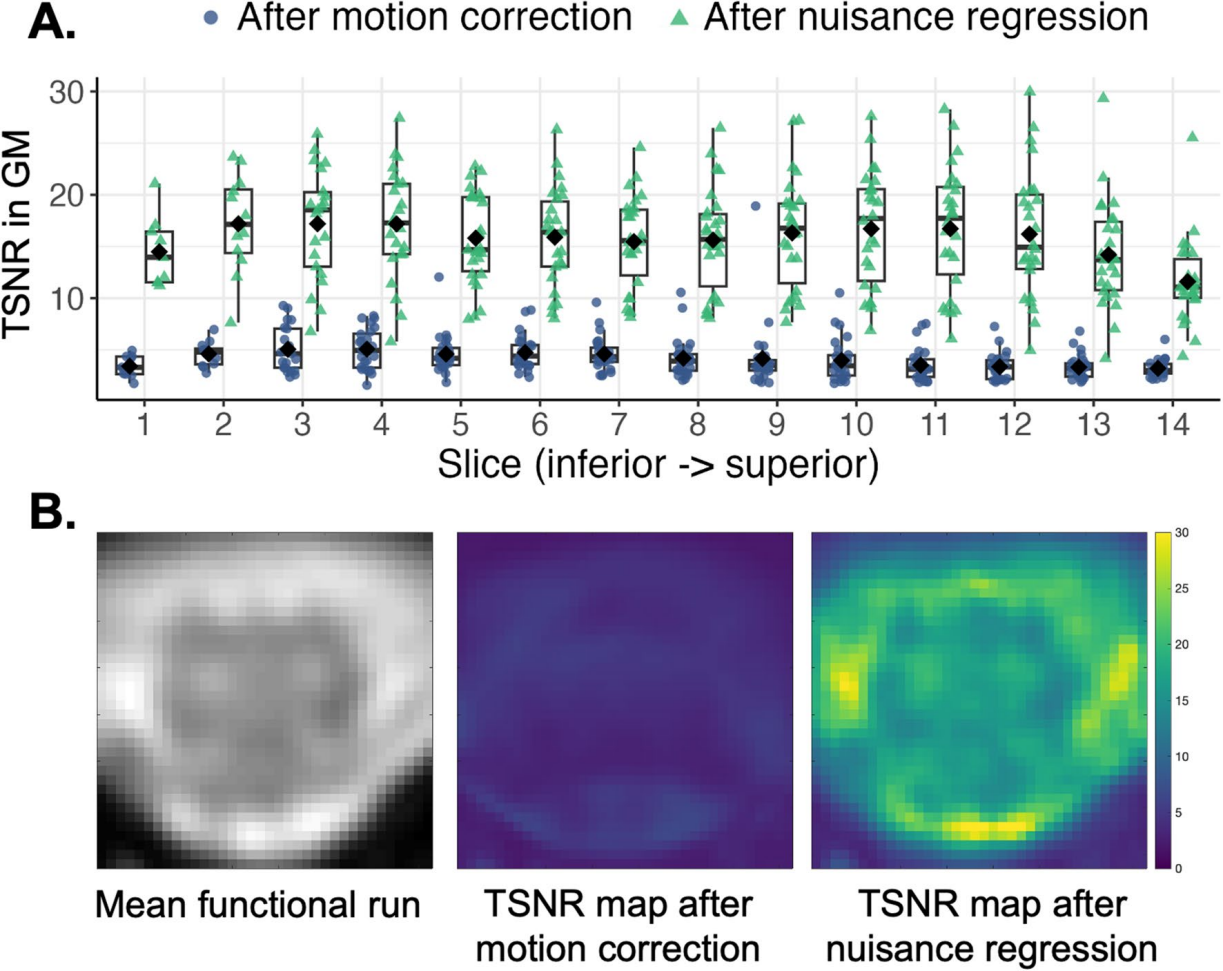
Temporal signal-to-noise ratio (TSNR). (**A**) Average gray matter (GM) TSNR by participant and slice after different processing steps for *n* = 26 participants. Boxplots show median and inter-quartile ranges, diamonds show group means. Note there are fewer data points for slices 1–3 as GM disappears in the lower cord below the conus medullaris. (**B**) TSNR maps for one example participant (43-year-old female) in one central slice.

Representative data are shown in Fig. 1. TSNR in the GM went from 4.32 ± 1.43 after motion correction to 16.35 ± 4.79 after denoising, a nearly fourfold increase (Fig. 2).

**Figure 3:**
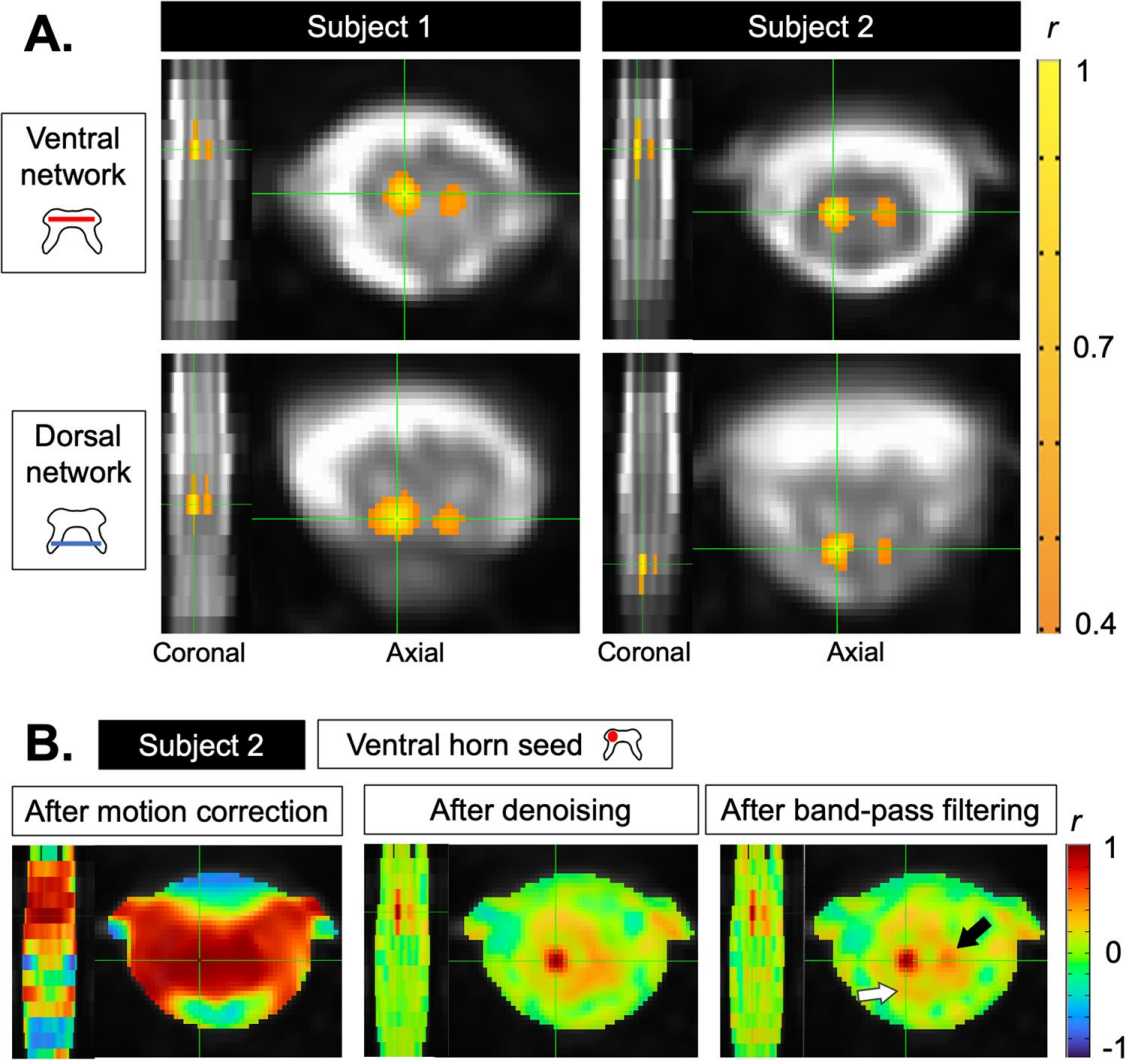
(**A**) Seed-based correlations in two participants (Subject 1: 27-year-old male, Subject 2: 32-year-old female) with AFNI-InstaCorr, thresholded at *r* = 0.4 (positive correlations only), uncorrected and un-clusterized, using a whole-cord (gray and white matter) mask on fully pre-processed data. Green crosshairs indicate each seed voxel. Slice locations are shown in the coronal plane to the left of each axial image. Top: positive correlations between a seed voxel in the left ventral horn and the contralateral horn, and the left ventral horn on adjacent slices. Bottom: positive correlations between a seed in the left dorsal horn and the contralateral dorsal horn, and the left dorsal horn in adjacent slices. (**B**) The same data as in the top right panel of A (Subject 2, ventral network) are shown at different stages of pre-processing, using a mask of the spinal canal (cord and cerebrospinal fluid (CSF)). Positive (yellow/red) and negative (blue) correlations are shown. Starting with a seed voxel in the left ventral horn, areas of high correlation are present across the cord within-slice and across several slices. After denoising, voxelwise correlations in the CSF revert towards zero (in green). After band-pass filtering, the contralateral ventral horn appears with further spatial precision (red region, black arrow). Moderate correlation can be seen in the ipsilateral dorsal horn (in orange, white arrow) that did not satisfy the threshold in the above top right panel. Similar maps for all networks and subjects at different stages of processing are shown in Supplemental Fig. S2.

Evidence of ventral and dorsal networks were observed in two representative participants using interactive visualization of seed-based correlations, with seed voxels placed in ventral and dorsal horns showing correlations with the contralateral region. Figure 3 shows examples of within- and between-slice correlations with similar appearance between the two participants (in-plane location, extent and intensity, although at different rostro-caudal levels). Similar results in six additional subjects are shown in Supplemental Fig. S1.

**Figure 4:**
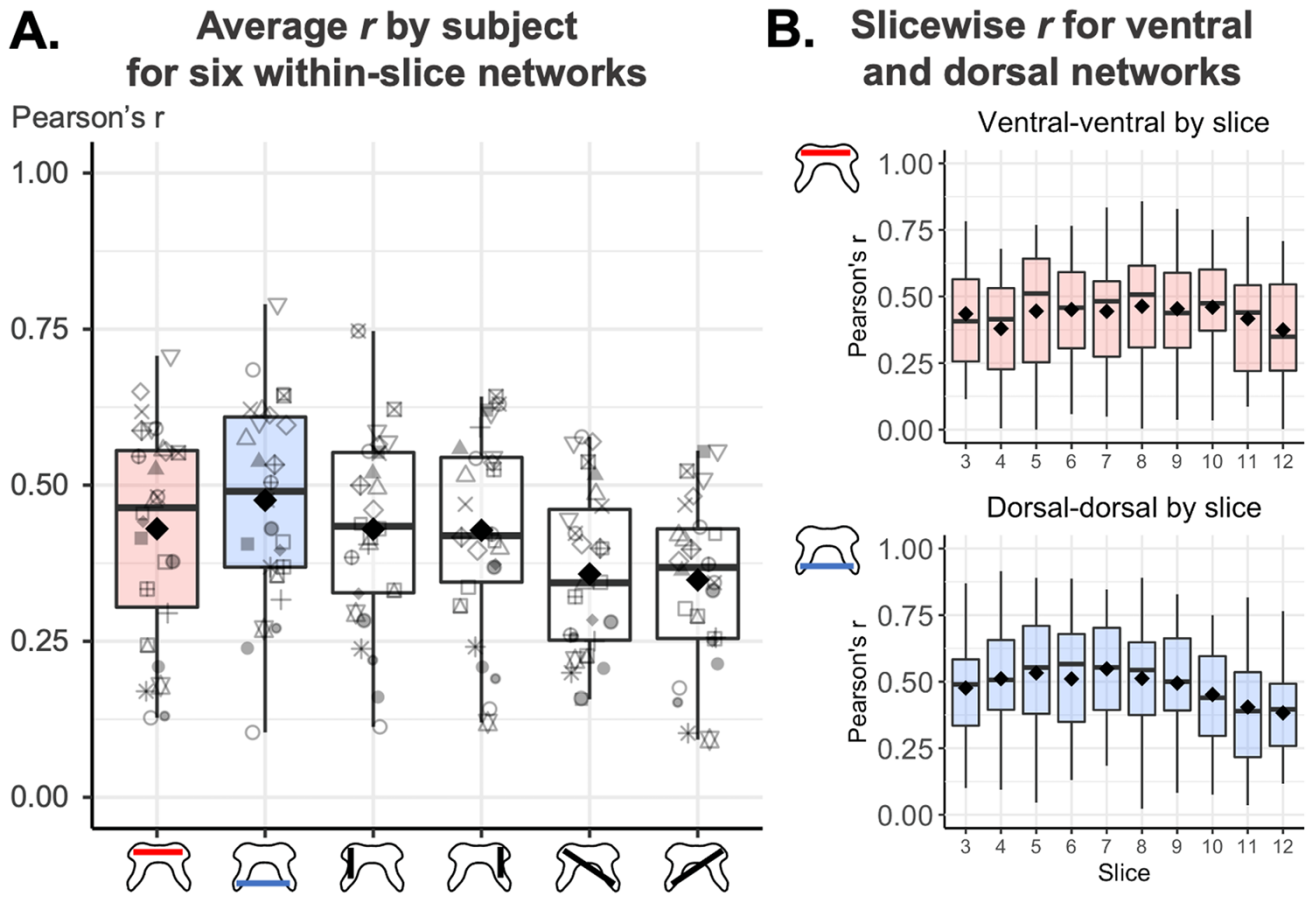
Within-slice connectivity (Pearson’s r) between different region pairs. Boxplots show median and inter-quartile ranges, diamonds show group means. Outliers are defined as laying beyond 1.5 times the inter-quartile range of either the first or third quartile. (**A**) Average r by participant and network for the central 10 slices. Shapes represent different participants. Average correlations between gray matter regions are: between ventral horns: 0.43 ± 0.17, between dorsal horns: 0.48 ± 0.16, between left ipsilateral ventral-dorsal: 0.43 ± 0.15, between right ipsilateral ventral-dorsal: 0.43 ± 0.16, between contralateral left ventral and right dorsal: 0.35 ± 0.13, between contralateral right ventral and left dorsal: 0.36 ± 0.13. Although the range in subject-average connectivity values is quite large (e.g. ~ 0.10–0.70 for the ventral network), there are no outlier data points. (**B**) Ventral and dorsal networks connectivity for the central 10 slices.

Correlation coefficients for each of six in-slice networks of interest are reported in Fig. 4. Group-average correlations were 0.43 ± 0.17 between the ventral horns, and 0.48 ± 0.16 between the dorsal horns.

### Independent component analysis

**Figure 5:**
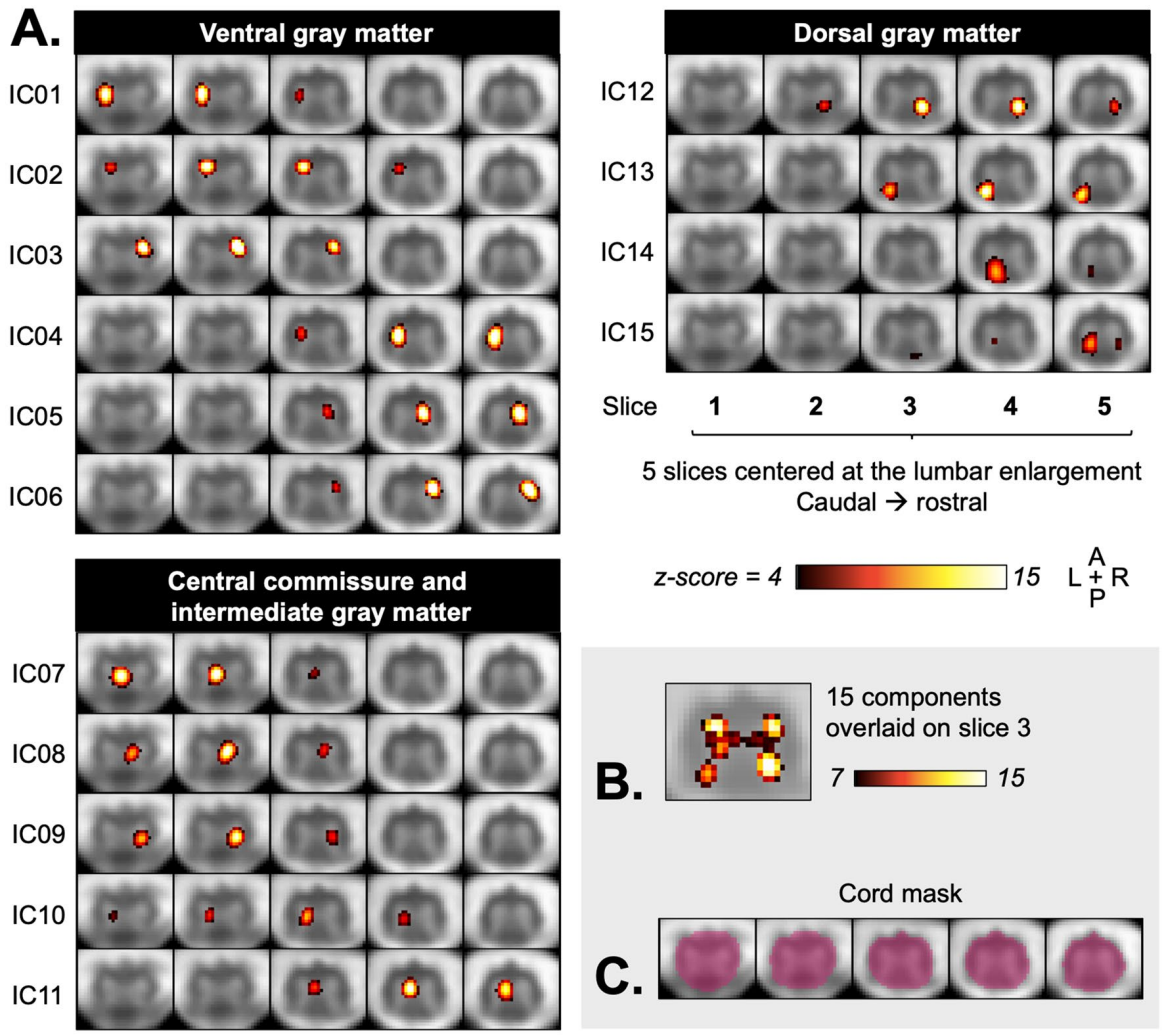
Group spatial independent component analysis (ICA)-derived components from 26 participants. (**A**) Aggregate group component maps are shown, overlaid on the group-average functional image, with each row showing one independent component across five slices (IC). Six ventral, 4 dorsal, and 5 central commissure/intermediate gray matter components are shown, organized top-to-bottom by their location from rostral to caudal and left to right. (**B**) Overlaid components for the middle slice (slice 3), thresholded at *z* ≥ 7, show the shape of the gray matter. (**C**) The cord mask used for analysis is shown in pink.

In the high-dimensionality ICA, fifteen GM (Fig. 5), 29 WM, and 6 noise-related ICA components (Supplemental Fig. S3) were identified. Six components were classified as ventral GM, 4 as dorsal, and 5 as central or intermediate GM. Components IC05 and IC12 are examples of neatly delineated ventral and dorsal GM components, respectively. Some components are ambiguously located at the boundary of GM and WM, and could belong to the latter (e.g. IC13, IC14). At the chosen threshold, all components were isolated to one side of the cord: 6 on the left, and 6 on the right, with a high degree of symmetry. Exceptions are IC07 and IC11 seemingly in the central commissure (although with a possible slight left-sidedness), and IC15 that displays bilateral activity in the dorsal horns on one slice. Most components extended across 3 slices (range: 2–4), and appeared segregated to either slices 1–3 or 3–5, with slice 3 having the appearance of a ‘transition’ zone between components above and below (e.g. components IC03 and IC06 located in the same area above and below slice 3, with some overlap). Unthresholded component maps, including negative correlations, are shown in Supplemental Fig. S4. A functional connectivity correlation matrix between the 15 obtained GM components is shown in Supplemental Fig. S5.

**Figure 6:**
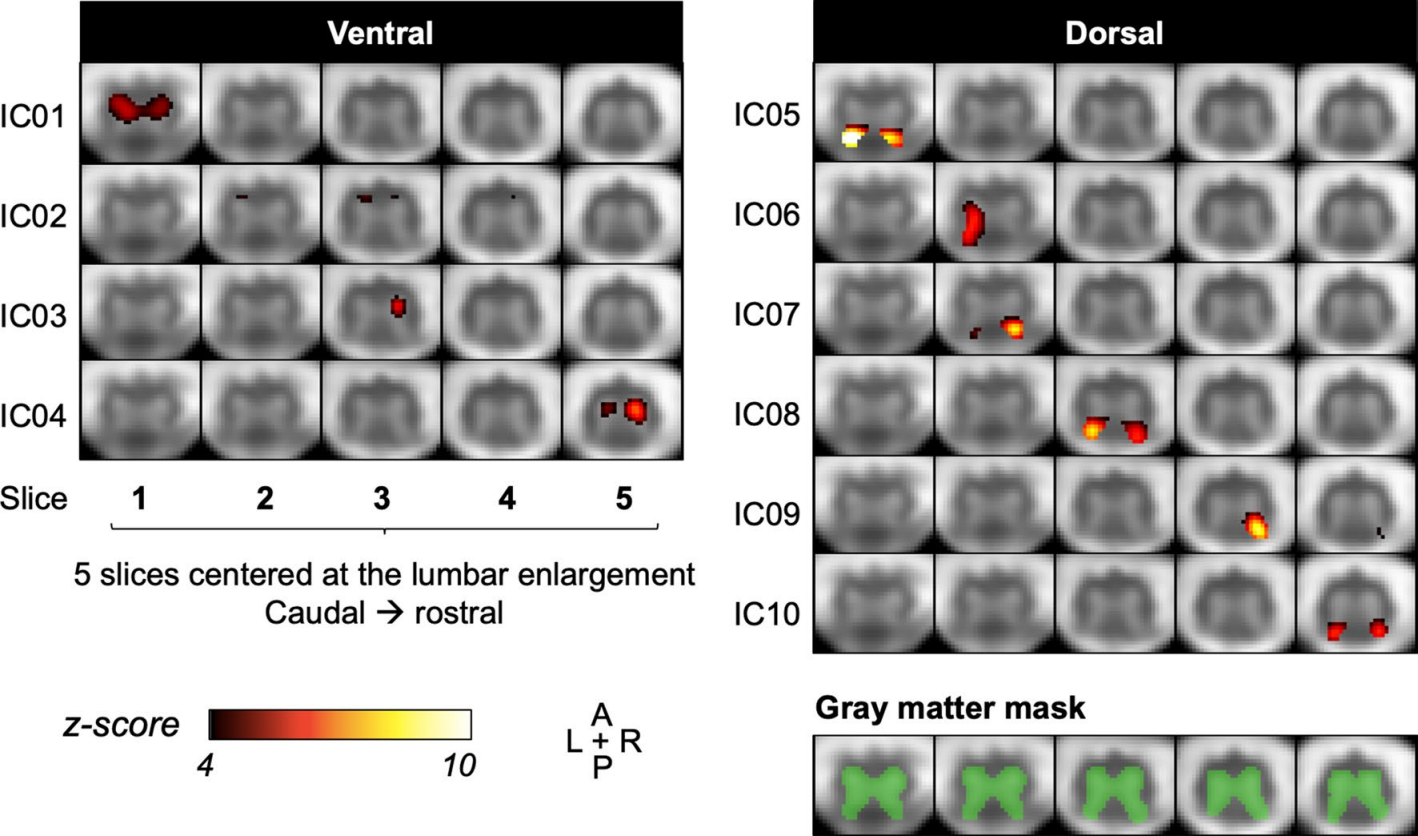
Group spatial independent component analysis (ICA) with gray matter (GM) mask (in green) and a preset of 10 components (low dimensionality). Aggregate group component maps are shown, overlaid on the group-average functional image, with each row showing one independent component across five slices. All 10 obtained components are shown: four components in the ventral GM, and six in the dorsal GM. At the same lower threshold chosen for the high-dimensionality ICA (*z* ≥ 4), bilateral fluctuations are seen in a majority of components.

In the lower-dimensionality ICA (Fig. 6), six bilateral components were observed: 2/4 in the ventral horns (IC01, IC04) and 4/6 in the dorsal horns (IC05, IC07, IC08, IC10). All bilateral components were constrained to one slice. IC03 and IC09 were unilateral ventral and dorsal horns components, respectively. Only one component, IC06, encompassed both ventral and dorsal regions unilaterally. Given its location at the mask edge, component IC02 is likely signal from white matter (partial volume effect) or noise. A connectivity correlation matrix for these 10 components is shown in Supplemental Fig. S5.

**Figure 7:**
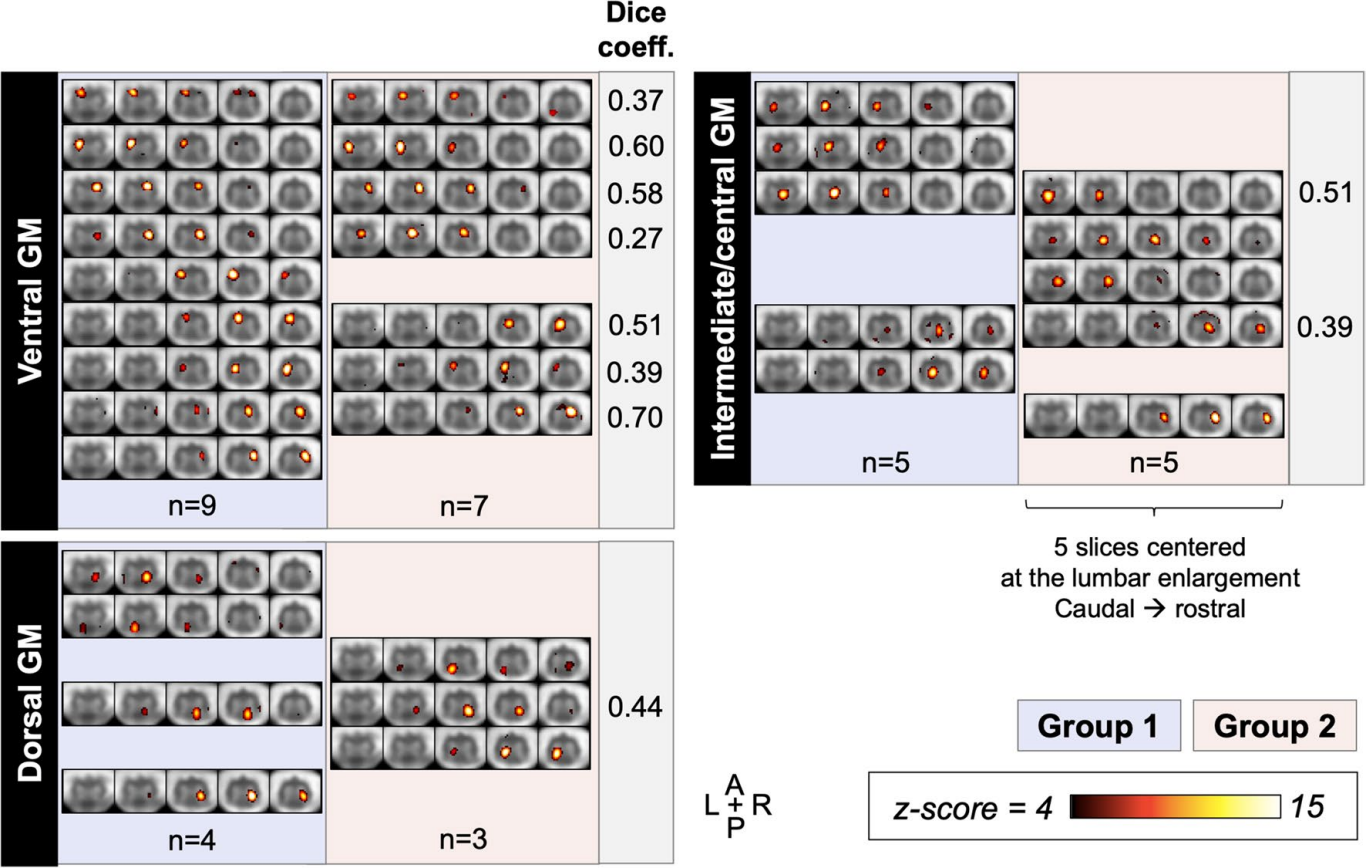
Independent component analysis (ICA) components in the gray matter (GM) from two demographically equivalent groups (each *n* = 13), with visually matched components displayed side-by-side along with corresponding Dice coefficients for spatial overlap. Components are classified into ventral, intermediate/central, and dorsal GM, and organized from caudal to rostral, and from left to right, with each row showing one component across five slices. Some components have no exact match. Note only components that could be confidently identified as GM were considered, to ensure rigorous matching.

In the split-half dataset analysis, TSNR was similar between sub-groups (see Supplemental Material). ICAs yielded 18 GM components for Group 1, and 15 for Group 2 (Fig. 7; see Supplemental Fig. S6 for WM and noise components). Components overall had the same appearance as those obtained with the full group analysis. All ventral, but fewer intermediate/central and dorsal components could be matched to a similar one between groups, although visual inspection showed a high degree of similarity between sub-groups when disregarding lateralization or exact rostro-caudal location. Average Dice coefficients were 0.48 across all GM components (range 0.27–0.70), and 0.50 across ventral GM components.

## Discussion

In this study, rs-fMRI data in the lumbar enlargement of the spinal cord were acquired in 26 healthy participants using a multi-shot GRE sequence, and functional connectivity was examined using seed- and region-based approaches and ICA. Temporal correlations reflecting functional connectivity were highest in the dorsal sensory and ventral motor networks, followed by ipsilateral and contralateral ventral-dorsal networks, similar to the pattern typically seen in the cervical spinal cord. Group spatial ICA revealed coherent activity in unilateral anterior, intermediate and posterior GM regions, reflecting the neuroanatomical organization of the spinal cord.

We observed good performance of the acquisition and analysis pipelines, as seen in the increase in TSNR and through visual data quality assessment at each step. TSNR in the GM after motion correction and denoising was on par with previously reported values for cervical cord rs-fMRI, ranging from ~ 15–20 across studies^22, 29^. The multi-shot GRE sequence used here has demonstrated sensitivity to hypercapnia-induced BOLD changes, as well as to a finger-tapping task^23^, and shown resting-state changes in the presence of pathology in multiple sclerosis at 3T^19^ and 7 T^18^. However, the sensitivity and reliability of different sequences in the lumbar cord specifically have not been evaluated. There may also be room for optimization of the current analysis methods for spinal cord rs-fMRI. Examples of possible analysis choices to evaluate include band-pass filtering range, systematic evaluation of the efficacy of removing cardiac and respiratory noise, and assessing the impact of including WM signal regression. Other possibilities to ensure data quality include motion ‘scrubbing’, or removal of individual dynamics beyond a chosen threshold for motion, a more stringent approach compared to the current one. Carpet plots may also show promise as a visual quality control method^30, 31^. Although carpet plots have only recently been implemented in the spinal cord^31^ and future exploration of their interpretation in this anatomical location is needed, examples of these plots are provided in Supplemental Fig. S7 to illustrate the effects of the nuisance removal. Such techniques may be implemented and their effects on signal quality and connectivity estimates evaluated.

Using region-based within-slice correlations, we observed the following pattern of network connectivity, in diminishing order of correlation strength: dorsal, followed by ventral, ventral-dorsal ipsilateral, and ventral-dorsal contralateral networks. Coherent activity in the ventral and dorsal horns is known to support motor and sensory function, respectively^4, 32–34^. Ventral network connectivity has consistently been observed to be the highest among studies reporting in-slice region-based correlations^18, 19, 22, 29, 33, 35–37^ and ICA-based connectivity^38^ in the cervical cord. Here, the average connectivity of the ventral network was lower than that of the dorsal network. This discrepancy could be due to inherently different characteristics of functional connectivity in the lumbar cord, or the greater amount of dorsal GM in this region compared to the cervical cord, facilitating detection of strong correlations. Beside this, the observed pattern of diminishing ipsilateral, followed by ventral-dorsal contralateral correlation strength is similar to that previously reported in cervical cord rs-fMRI studies^22, 29^.

Adequately removing all noise sources is paramount to ensuring the observed signal fluctuations correspond to BOLD signal and are not artefactual or a byproduct of other physiological phenomena. In addition to RETROICOR for physiological denoising, we used a component-based nuisance removal method as recommended for rs-fMRI in the cervical cord^37, 39, 40^ which includes regression of slicewise signal components from the CSF (see Fig. 1) and across the whole ‘not-cord’ FOV (i.e. entire axial slice minus the spinal canal). WM signal was not removed, due to: (1) our own and others’ observations that this step has no impact on results^40^, (2) the risk of removing GM signal of interest due to partial volume effects in those small areas, and (3) the notion that there may be BOLD or BOLD-related signal in WM that may be biologically meaningful and related to GM signal of interest^41^. Finally, while global signal of non-neuronal origin affecting all GM voxels is a concern in brain fMRI studies, previous work has shown its removal is not required for SC rs-fMRI data. Studies in the cervical SC attempted to control for this when reporting in-slice correlations between regions by regressing out signal from other GM ROIs in-slice (e.g. reporting functional connectivity in the ventral network by reporting the partial correlation of the two ventral horns with average time-series from the two dorsal horns as covariates). In those three separate experiments, partial and bivariate correlations (as employed here) led to similar results^37, 39, 40^. Moreover, here, a seed-based voxelwise approach showed highest correlations between bilateral horns, and to a lesser extent with the rest of the GM in-slice. Those observations of localized synchronous activity following adequate pre-processing in the expected functional networks (ventral and dorsal) instead of all adjacent GM suggests they are originating from distinct functional areas and not simply a byproduct of breathing or a global signal phenomenon. However, despite these considerations, it is possible that some noise may persist in the GM despite thorough noise removal steps ^40^. To further ascertain the neuronal origin of the observed fluctuations, other analyses of the time-series themselves could be performed, e.g. in the frequency domain.

Using group spatial ICA, we observed components that could be classified into ventral, dorsal, and intermediate/central GM. Here, each individual ventral and dorsal component from the main ICA analysis had a contralateral counterpart, consistent with the existence of bilateral networks in support of motor and sensory function^4, 32–34^. Fewer components were observed in the dorsal compared to the ventral GM, and all dorsal components were in the upper slices. The proportion of GM decreases when moving caudally; moreover, inter-subject registration of the dorsal horns may not be as effective due to their anatomy, making detection of dorsal components more difficult, as also noted by Kinany et al.^42^. Refinements of inter-subject registration to improve detection of GM networks could be aided by the creation and use of a template to leverage existing tools for standard-space analysis, since to the authors’ knowledge, the only currently available T_2_^*^-weighted spinal cord template (PAM50 within SCT) only extends down to the T12 vertebral level^5^.

Observation of the main ICA output revealed several components located in the intermediate region, as well as in the central commissure (with the present criteria, 5/16 GM components). Most previous uses of ICA with cervical cord rs-fMRI data showed networks that overlapped both GM and WM, and generally showed bilateral activity, but were only classified into ventral and dorsal clusters^38, 43, 44^. There are limited reports of coherent signal fluctuations attributed to regions other than anterior and posterior GM in cervical cord rs-fMRI studies, and intermediate and central GM have seldom been explicitly examined. Using the central commissure as a predetermined ROI, Vahdat et al. found significant connectivity with several bilateral brain regions that were consistent with its role in supporting sensorimotor processing and connecting the hemicords^44^. Kong et al. noted the presence of centrally located components, which they classified as being ‘of no interest’ and attributed to CSF flow in the central canal^38^. San Emeterio Nateras et al. also observed midline GM components, and proposed those could reflect the activity of inter-neuronal synapses in the cytoarchitectonically distinct central GM^45^. Here, the size and spatial extent of the central components point to them originating in GM rather than the smaller CSF-filled central canal. The denoising process used here was also potentially more thorough than that used in the earlier paper by Kong et al., which used a single regressor from the CSF and cord region, compared to the present approach where denoising was performed slicewise and relied on regression of several components from principal component analysis of the signal in a CSF mask. Using a dynamic functional connectivity approach, Kinany et al. found GM components in the ventral and intermediate, but not dorsal, regions^42^. The intermediate GM column contains neurons whose function relates to the motor and sensory role of the ventral and dorsal regions, and also supports autonomic function in the upper lumbar cord^4, 34^. Description of the functional connectivity of those regions using a region-based approach would be of interest in both the cervical and lumbar cord.

Despite the limited superior-inferior field of view, most GM components extended across 3–4 slices. Since inter-subject registration was done in-plane, any blurring due to interpolation would not occur in the *z* direction; this is a strong sign that coherent neuronal fluctuations extend, and can be detected, rostro-caudally. Functional networks have been shown to exhibit segmental organization in the cervical cord^38, 42, 45, 46^. Here, manual positioning of the acquisition volume at the center of the lumbar enlargement was used, as lumbar spinal levels are not aligned with vertebral levels. Thus, the enlargement itself as identifiable on a sagittal T_2_ scan was chosen as a landmark rather than proximity to particular vertebral bodies^5^. Inter-subject alignment could be achieved by labeling slices relative to that with the largest calculated cross-sectional area^47, 48^. Future research could evaluate inter-slice or inter-level connectivity, for instance using the two regions identified with ICA as forming seemingly distinct rostro-caudal networks as a starting point for defining ROIs.

The choice of the number of ICA components is known to influence output^28^. As such, some areas showing closely synchronous signal fluctuations may be ‘split’ during analysis (e.g. IC05 and IC06 in the high dimensionality ICA (Fig. 5), which both appear clearly situated within the right ventral GM across the same slices). Conversely, IC15 shows bilateral activity in the dorsal horns, bearing in mind ROI-based analysis showed the highest correlations between contralateral dorsal horns. As expected, lower-dimensionality ICA in the GM led to an output with a majority of bilateral components corresponding to ventral and dorsal networks (Fig. 6). More bilateral components with stronger amplitude were seen in the dorsal horns than in ventral horns, consistent with the fact that dorsal-dorsal correlations are the highest among region-pairs when using a region-based approach. Kinany et al. similarly observed that specifying a higher number of components led to higher ‘granularity’ in ICA output, while a lower input number created bilateral components^42^. The degree of connectivity and interdependence of components could be evaluated in future analyses by examining their temporal inter-correlations, as shown with an example correlation matrix in Supplemental Fig. S5, or further varying the input number of components for ICA. On the other hand, and as noted above, functional parcellation of GM beyond the four horns and central commissure is an interesting area of development, and such segregation could reflect the activity of neighboring but functionally distinct neuronal populations^34, 49^.

Finally, a large number of ICA components were seen in the WM, likely due to its larger size compared to GM. There is mounting evidence that BOLD fluctuations in the cerebral WM can be detected and are functionally relevant, whether attributable to metabolic activity in the WM itself in support of neuronal function, or driven by the vascular response to GM neuronal activity^41^. Such components have been shown in the cervical cord to reflect the organization of WM tracts^41, 42^ and may present specific functional connectivity patterns to other WM regions^36^, which will be investigated in future work.

Regarding sub-group ICA, qualitative consistency between several components was observed, particularly in the ventral GM. While perfect overlap is not expected between distinct participant groups, Dice coefficients reflected moderate overlap. More GM components were obtained in Group 1; since TSNR was similar between groups, this could be due to differences in quality of registration or intrinsic inter-subject differences. Inclusion of more participants will enable a quantitative evaluation of the stability of networks across participants and sessions, with intra- and inter-session reliability experiments underway. Methods like group-information guided ICA ^50^ or dual regression would allow the selection of consistent components between datasets and direct comparison of their features, including functional connectivity between components. Interestingly, some WM and noise components showed high spatial correspondence between sub-groups, which also merits further investigation. The qualitative consistency between observed components in the two separate sub-groups points to the robustness of the current approach and should be quantified using a larger or longitudinal dataset.

Some degree of uncertainty in the localization of components exists due to the subjectivity inherent to visual classification. Strategies to increase confidence in component localization include calculating the overlap of thresholded component maps with a GM mask or anatomical atlas. Other limitations include the possible dependance of numerical results on the methodological choices described above, as well as the qualitative approach to independent component identification, which can be addressed by replication of the present results by other groups, and via additional analysis approaches as mentioned above, respectively.

This study demonstrates successful implementation of a rs-fMRI acquisition protocol and analysis pipeline and provides information on functional network connectivity in the lumbar cord using both hypothesis- and data-driven methods. This work serves as a starting point for exploration of the functional properties of the lumbar spinal cord. Future work will investigate the reliability of measures obtained with seed-based and data-driven approaches. Rs-fMRI of the lumbar cord may offer further opportunities for investigations into clinical populations with neurological dysfunction or injury.

### Supplementary Information

Supplementary Information.

## Data Availability

The datasets generated during this study are available from the corresponding author on reasonable request.

## References

[1] Powers JM, Ioachim G, Stroman PW (2018). Ten key insights into the use of spinal cord fMRI. Brain Sci..

[2] Weier K (2012). Biplanar MRI for the assessment of the spinal cord in multiple sclerosis. Mult. Scler. J..

[3] van Den Hauwe, L., Sundgren, P.C. & Flanders, A.E. Spinal Trauma and Spinal Cord Injury (SCI). in Diseases of the Brain, Head and Neck, Spine 2020–2023 (eds. Hodler, J., Kubik-Huch, R. A. & von Schulthess, G. K.) 231–240 (Springer International Publishing, 2020). doi:10.1007/978-3-030-38490-6_19 32119240

[4] Cho TA (2015). Spinal cord functional anatomy. Contin. Lifelong Learn. Neurol..

[5] De Leener B (2018). PAM50: Unbiased multimodal template of the brainstem and spinal cord aligned with the ICBM152 space. Neuroimage.

[6] Dehghani H, Oghabian MA, Batouli SAH, Kheradmand JA, Khatibi A (2020). Effect of physiological noise on thoracolumbar spinal cord functional magnetic resonance imaging in 3T magnetic field. Basic Clin. Neurosci..

[7] Figley CR, Yau D, Stroman PW (2008). Attenuation of lower-thoracic, lumbar, and sacral spinal cord motion: implications for imaging human spinal cord structure and function. Am. J. Neuroradiol..

[8] Stroman PW, Tomanek B, Krause V, Frankenstein UN, Malisza KL (2002). Mapping of neuronal function in the healthy and injured human spinal cord with spinal fMRI. Neuroimage.

[9] Stroman PW (2004). Noninvasive assessment of the injured human spinal cord by means of functional magnetic resonance imaging. Spinal Cord.

[10] Lawrence JM, Stroman PW, Kollias SS (2008). Functional magnetic resonance imaging of the human spinal cord during vibration stimulation of different dermatomes. Neuroradiology.

[11] Kornelsen J (2013). Functional MRI of the thoracic spinal cord during vibration sensation. J. Magn. Reson. Imag..

[12] Kornelsen J, Stroman PW (2004). fMRI of the lumbar spinal cord during a lower limb motor task. Magn. Reson. Med..

[13] Alexander M, Kozyrev N, Figley CR, Richards JS (2017). Altered spinal cord activity during sexual stimulation in women with SCI: a pilot fMRI study. Spinal Cord Ser. Cases.

[14] Kashkouli Nejad, K. et al. Spinal fMRI of interoceptive attention/awareness in experts and novices. Neural Plast **2014**, 6–9 (2014) 10.1155/2014/679509 PMC4086226 25031872

[15] Kornelsen J, Smith SD, McIver TA (2013). A neural correlate of visceral emotional responses: Evidence from fMRI of the thoracic spinal cord. Soc. Cogn. Affect. Neurosci..

[16] Combes, A.J.E. et al. Detection of resting-state functional connectivity networks in the human lumbar spinal cord at 3T. in Proc. Int. Soc. Magn. Reson. Med. (2022)

[17] Ricchi, I., Kinany, N. & Van De Ville, D. Resting-state fMRI of the lumbar spine: Static and dynamic functional connectivity. in Proc Hum. Brain Mapp. (2022)

[18] Conrad BN (2018). Multiple sclerosis lesions affect intrinsic functional connectivity of the spinal cord. Brain.

[19] Combes AJE (2022). Functional connectivity in the dorsal network of the cervical spinal cord is correlated with diffusion tensor imaging indices in relapsing-remitting multiple sclerosis. Neuroimage Clin..

[20] Landelle C (2023). Altered spinal cord functional connectivity associated with Parkinson’s disease progression. Mov. Disord..

[21] Kinany N (2022). Towards reliable spinal cord fMRI: Assessment of common imaging protocols. Neuroimage.

[22] Barry RL, Conrad BN, Smith SA, Gore JC (2018). A practical protocol for measurements of spinal cord functional connectivity. Sci. Rep..

[23] Barry RL (2021). Multi-shot acquisitions for stimulus-evoked spinal cord BOLD fMRI. Magn. Reson. Med..

[24] Smith SM (2004). Advances in functional and structural MR image analysis and implementation as FSL. Neuroimage.

[25] De Leener B (2017). SCT: Spinal cord toolbox, an open-source software for processing spinal cord MRI data. Neuroimage.

[26] Glover GH, Li T-Q, Ress D (2000). Image-based method for retrospective correction of physiological motion effects in fMRI: RETROICOR. Magn. Reson. Med..

[27] Song S (2017). Temporal similarity perfusion mapping: A standardized and model-free method for detecting perfusion deficits in stroke. PLoS One.

[28] Calhoun VD, Adali T, Pearlson GD, Pekar JJ (2001). A method for making group inferences from functional MRI data using independent component analysis. Hum. Brain. Mapp..

[29] Eippert F (2017). Investigating resting-state functional connectivity in the cervical spinal cord at 3T. Neuroimage.

[30] Power JD (2017). A simple but useful way to assess fMRI scan qualities. Neuroimage.

[31] Hemmerling, K.J. & Bright, M.G.A visualization tool for assessment of spinal cord functional magnetic resonance imaging data quality. in 2021 43rd Annual International Conference of the IEEE Engineering in Medicine & Biology Society (EMBC) 3391–3394 (IEEE, 2021). 10.1109/EMBC46164.2021.9630903 10.1109/EMBC46164.2021.9630903 34891967

[32] Landelle C (2021). Investigating the human spinal sensorimotor pathways through functional magnetic resonance imaging. Neuroimage.

[33] Weber KA (2018). Thermal stimulation alters cervical spinal cord functional connectivity in humans. Neuroscience.

[34] Altman, J. & Bayer, S. A. Development of the human spinal cord: An interpretation based on experimental studies in animals. (Oxford University Press, 2001)

[35] Harita S, Stroman PW (2017). Confirmation of resting-state BOLD fluctuations in the human brainstem and spinal cord after identification and removal of physiological noise. Magn. Reson. Med..

[36] Barry RL, Smith SA, Dula AN, Gore JC (2014). Resting state functional connectivity in the human spinal cord. Elife.

[37] Barry RL, Rogers BP, Conrad BN, Smith SA, Gore JC (2016). Reproducibility of resting state spinal cord networks in healthy volunteers at 7 Tesla. Neuroimage.

[38] Kong Y (2014). Intrinsically organized resting state networks in the human spinal cord. Proc. Natl. Acad. Sci. USA.

[39] Eippert F, Kong Y, Jenkinson M, Tracey I, Brooks JCW (2017). Denoising spinal cord fMRI data: Approaches to acquisition and analysis. Neuroimage.

[40] Kaptan M (2023). Reliability of resting-state functional connectivity in the human spinal cord: Assessing the impact of distinct noise sources. Neuroimage.

[41] Gore JC (2019). Functional MRI and resting state connectivity in white matter—a mini-review. Magn. Reson. Imag..

[42] Kinany N, Pirondini E, Micera S, Van De Ville D (2020). Dynamic functional connectivity of resting-state spinal cord fmri reveals fine-grained intrinsic architecture. Neuron.

[43] Wei P (2010). Resting state networks in human cervical spinal cord observed with fMRI. Eur. J. Appl. Physiol..

[44] Vahdat S (2020). Resting-state brain and spinal cord networks in humans are functionally integrated. PLoS Biol.

[45] San Emeterio Nateras O, Yu F, Muir ER, Bazan C, Franklin CG, Li W, Li J, Lancaster JL, Duong TQ (2016). Intrinsic resting-state functional connectivity in the human spinal cord at 30 T. Radiology..

[46] Vahdat S (2015). Simultaneous brain-cervical cord fMRI reveals intrinsic spinal cord plasticity during motor sequence learning. PLoS Biol.

[47] Yiannakas MC (2019). Gray vs. white matter segmentation of the conus medullaris: Reliability and variability in healthy volunteers. J. Neuroimag..

[48] Büeler S (2022). Optimized multi-echo gradient-echo magnetic resonance imaging for gray and white matter segmentation in the lumbosacral cord at 3T. Sci. Rep..

[49] Sengupta A (2021). Functional networks in non-human primate spinal cord and the effects of injury. Neuroimage.

[50] Du Y, Fan Y (2013). Group information guided ICA for fMRI data analysis. Neuroimage.

