# Supplementary material for: Detection of resting-state functional connectivity in the lumbar spinal cord with 3T MRI
Source: Sci Rep. 2023 Oct 24;13:18189. doi: 10.1038/s41598-023-45302-0 (PMC10597994; doi:10.1038/s41598-023-45302-0)
Supplement: Supplementary file 1 — Supplementary Information. [file 41598_2023_45302_MOESM1_ESM.pdf]

Detection of Resting-State Functional Connectivity in the Lumbar Spinal Cord with 3T MRI

Authors:
Anna Combes[1,2], Lipika Narisetti[1], Anirban Sengupta[1,2], Baxter P. Rogers[1,2], Grace Sweeney[1], Logan Prock[1], Delaney Houston[1], Colin D. McKnight[2], John C. Gore[1,2,3], Seth A. Smith[1,2,3], Kristin P. O'Grady[1,2,3,*]

Affiliations:
[1]Vanderbilt University Institute of Imaging Science, Vanderbilt University Medical Center, Nashville, TN 37232, USA
[2]Department of Radiology and Radiological Sciences, Vanderbilt University Medical Center, Nashville, TN 37232, USA
[3]Department of Biomedical Engineering, Vanderbilt University, Nashville, TN 37235, USA

***Corresponding author:**
Prof. Kristin P. O'Grady
Email: kristin.p.ogrady@vumc.org
Corresponding address: 1161 21st Ave S, MCN AA1105, Nashville, TN 37232, USA
Tel: 615-322-7209

SUPPLEMENTAL MATERIAL

Demographic breakdown of sub-groups for independent component analysis

Group 1 (n=13): 7 females, 6 males, mean age 39.2±14.4, temporal signal-to-noise ratio (TSNR): 16.42±5.28

Group 2 (n=13): 8 females, 5 males, mean age 39.8±10.3, TSNR: 16.28±4.46

The groups did not significantly differ in age or TSNR (t-tests: $p>.05$).

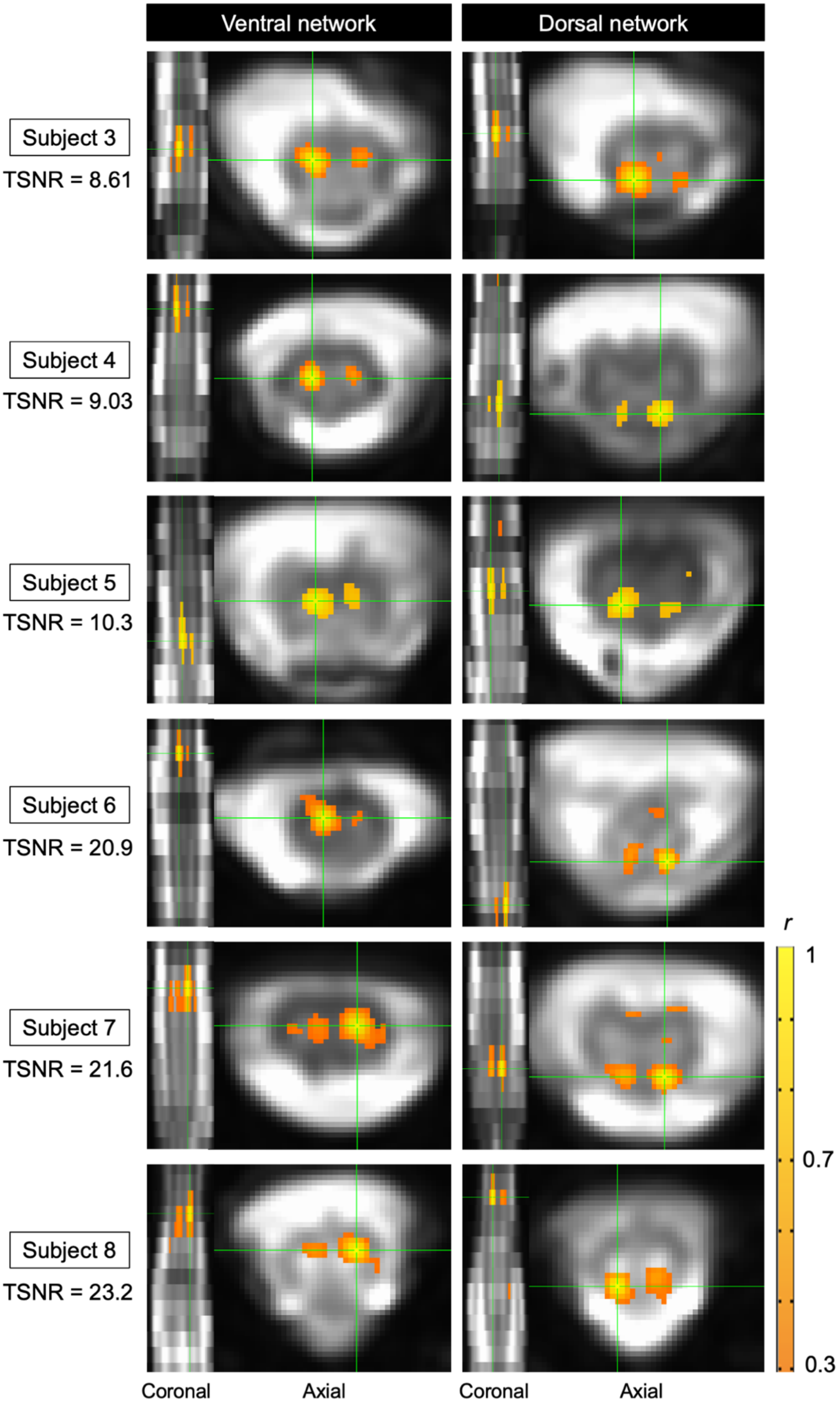

Ventral network
Dorsal network
Subject 3
TSNR = 8.61
Subject 4
TSNR = 9.03
Subject 5
TSNR = 10.3
Subject 6
TSNR = 20.9
Subject 7
TSNR = 21.6
Subject 8
TSNR = 23.2
Coronal
Axial
Coronal
Axial
r
1
0.7
0.3

Supplemental Figure S1. Seed-based correlations for six subjects with a range of temporal signal-to-noise ratio (TSNR), with seeds in the ventral and dorsal gray matter horns. Maps were obtained by interactive visualization in AFNI-InstaCorr with a threshold of r=0.3 and a whole-cord mask. (Note the threshold is lower than that used for the two subjects in **Figure 5**, to obtain good visualization across this sample.)

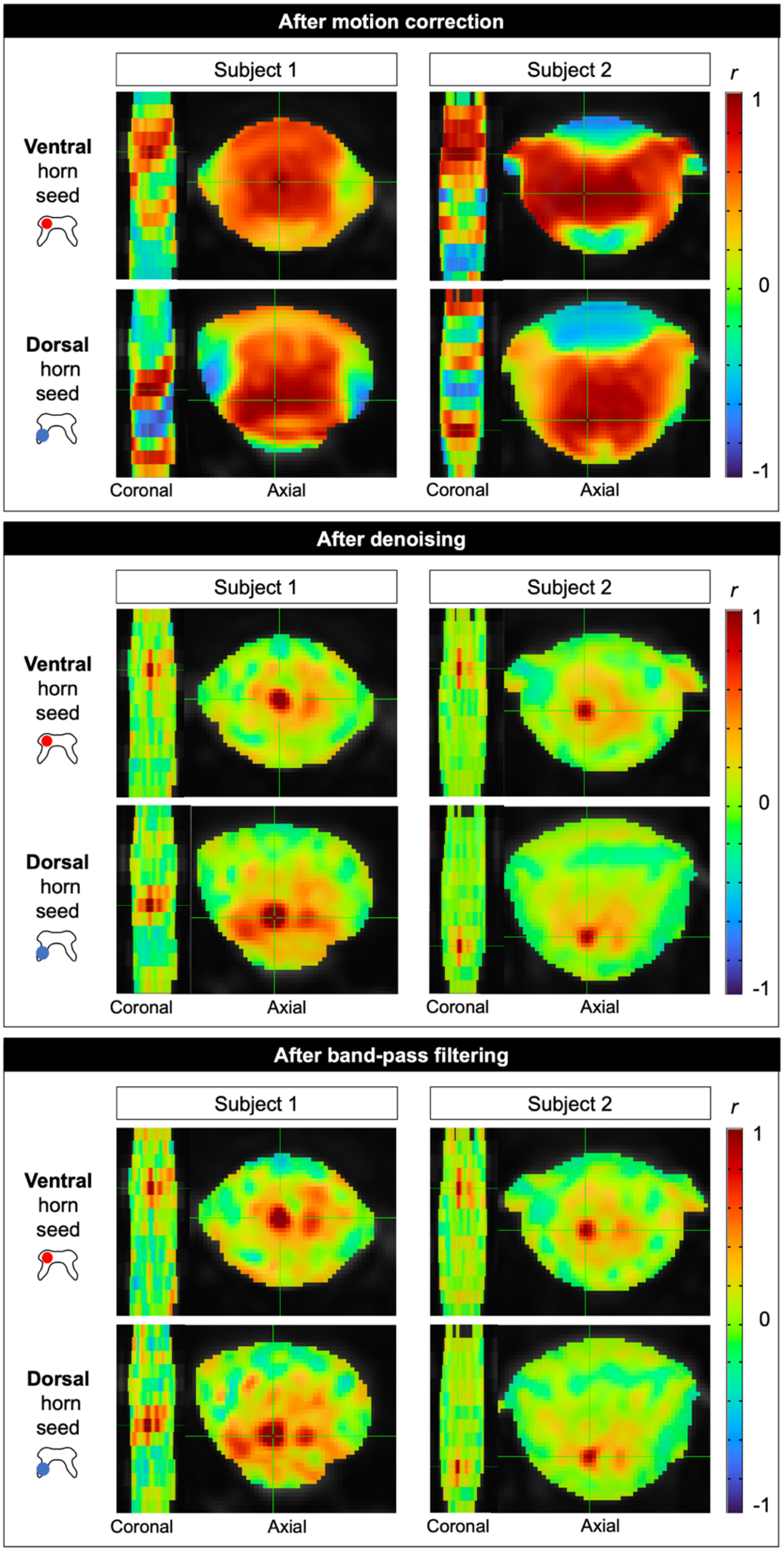
After motion correction
Subject 1
Subject 2
r
1
0
-1
Ventral horn seed
Dorsal horn seed
Coronal
Axial
Coronal
Axial
After denoising
Subject 1
Subject 2
r
1
0
-1
Ventral horn seed
Dorsal horn seed
Coronal
Axial
Coronal
Axial
After band-pass filtering
Subject 1
Subject 2
r
1
0
-1
Ventral horn seed
Dorsal horn seed
Coronal
Axial
Coronal
Axial

Supplemental Figure S2. Seed-based correlations in the same two participants as **Figure 3** (Subject 1: 27-year-old male, Subject 2: 32-year-old female) obtained with AFNI-InstaCorr, unthresholded and showing both positive and negative correlations, with a spinal canal mask. The three panels show voxelwise correlations with the same seed voxel (green crosshairs) at different stages of pre-processing. Top: after motion correction, middle; after denoising, bottom: after temporal band-pass filtering. Within each panel, the four images show the same two subjects each with a seed in a ventral, and a dorsal horn region. After denoising, signal correlations with cerebrospinal fluid and white matter voxels are lower, and positive correlations localized to gray matter are revealed. Band-pass filtering appears to only have a small effect, enhancing the spatial specificity of areas showing synchronous signal. Note that correlation strength between gray matter horns does not depend on spatial proximity: each seed region shows stronger correlations with the contralateral region (ventral to ventral, dorsal to dorsal) than with the ipsilateral region despite contiguity.

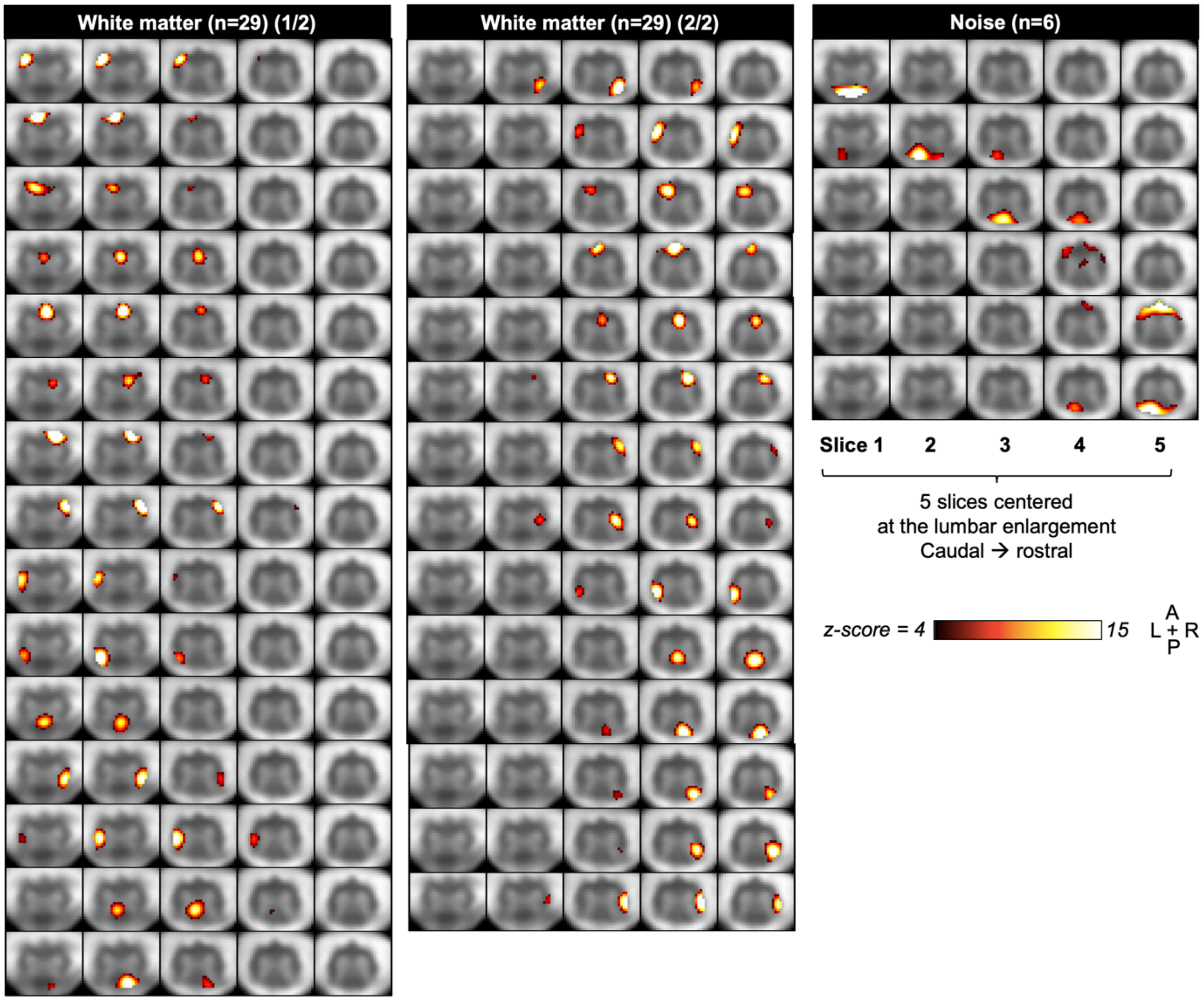

Supplemental Figure S3. White matter (WM) and noise components from group spatial independent component analysis (ICA) in 26 participants. Aggregate group component maps are shown overlaid on the group-average functional image, with each row showing one component across five slices. Components are organized from caudal to rostral, and from left to right. Note there is some degree of uncertainty in the localization of components due to the subjectivity inherent to visual classification.

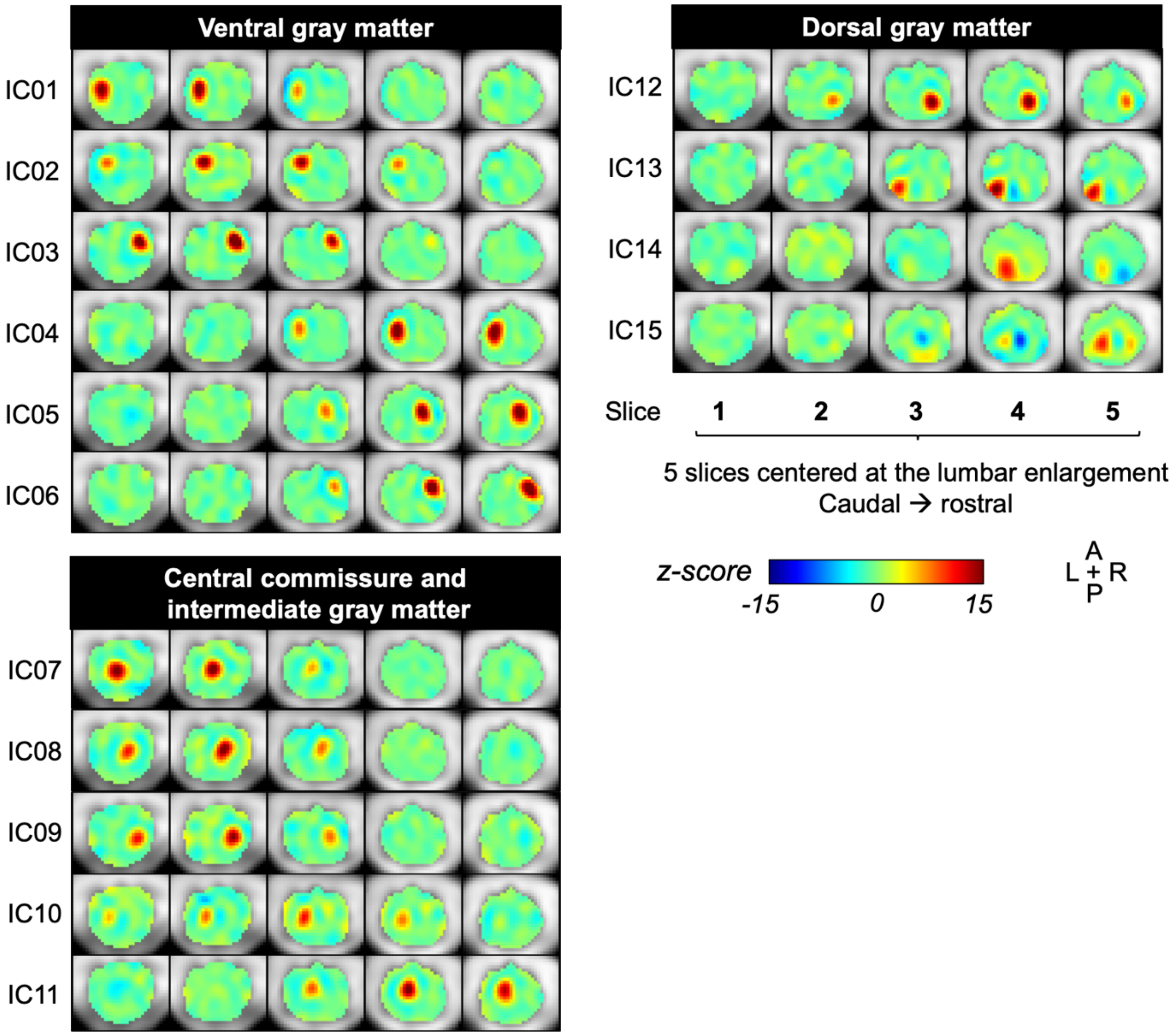

Supplemental Figure S4. Group spatial independent component analysis (ICA)-derived components from 26 participants. The same data and layout as **Figure 5** are shown unthresholded and including negative correlations (-15 ≤ z ≤15). The nodes identified in the thresholded maps have neat spatial delineation. A few components show some apparently anti-correlated areas, e.g. IC07, IC14 and IC15 showing negative correlations of one dorsal region with the contralateral dorsal region on an adjacent slice. Some components also show negative correlations in the same region on distal slices, components IC05, IC08, IC09 and IC11.

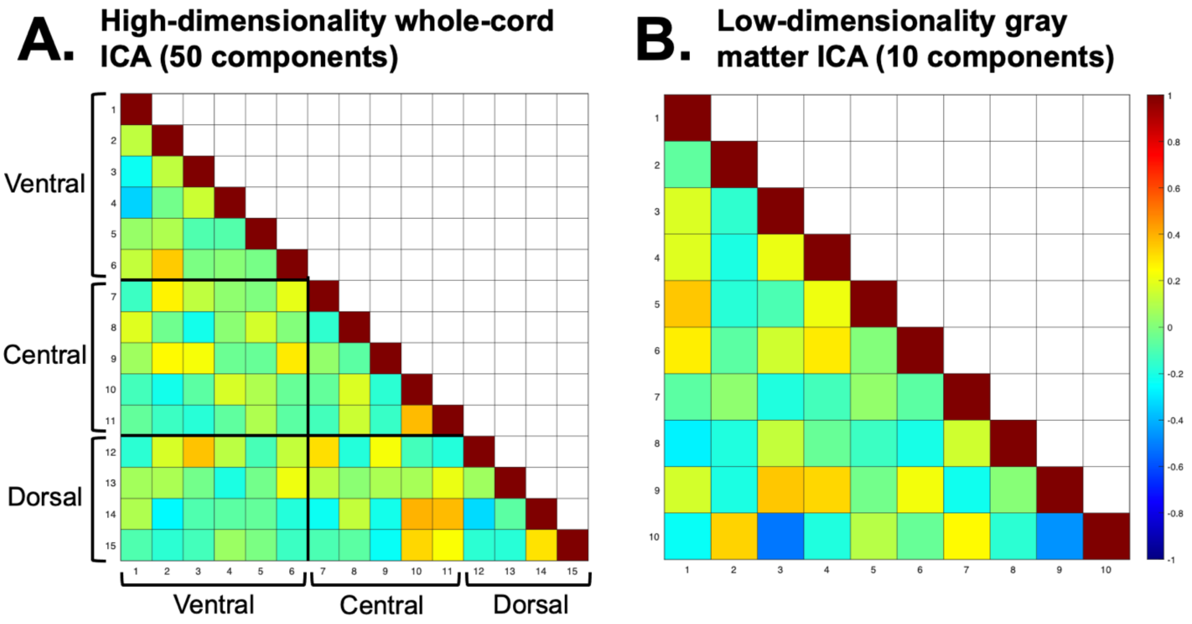

Supplemental Figure S5. Correlation matrices between independent component analysis (ICA) gray matter (GM) components. Pairwise Pearson's correlations are calculated between time-courses extracted from group-level components. A) Correlations between the fifteen GM components obtained with high-dimensionality ICA. B) Correlations between the 10 components in the low-dimensionality GM ICA.

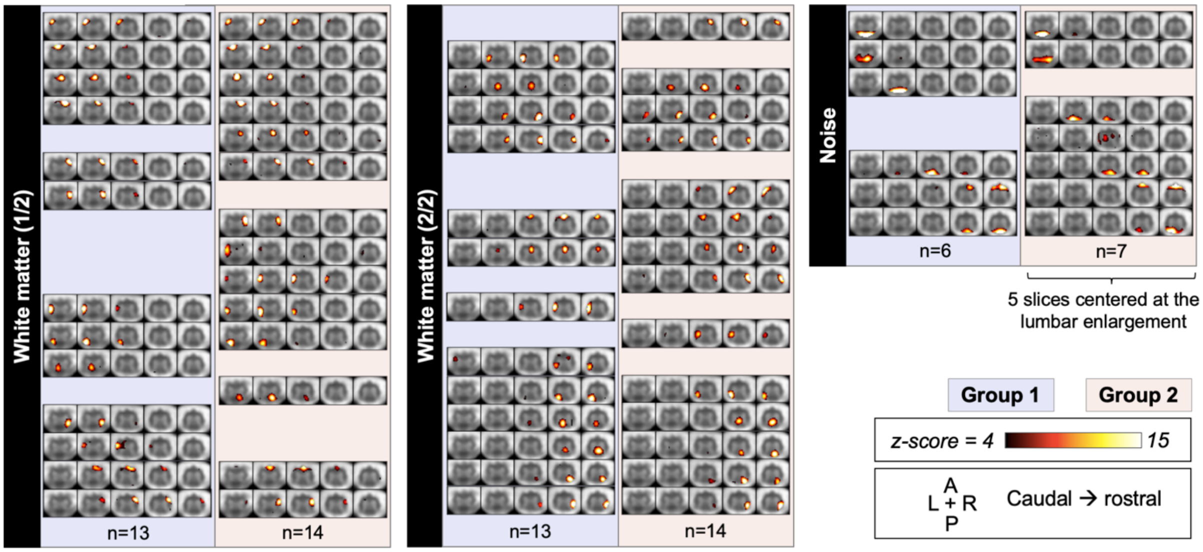

Supplemental Figure S6. Remaining components from the separate sub-group independent component analyses (ICA). Visually matched components belonging to white matter and identified as noise-related are displayed side-by-side, with each row showing one independent component across five slices. Components are organized from caudal to rostral, and from left to right, and shown overlaid on the group-average functional image. Note there is some degree of uncertainty in the localization of components due to the subjectivity inherent to visual classification.

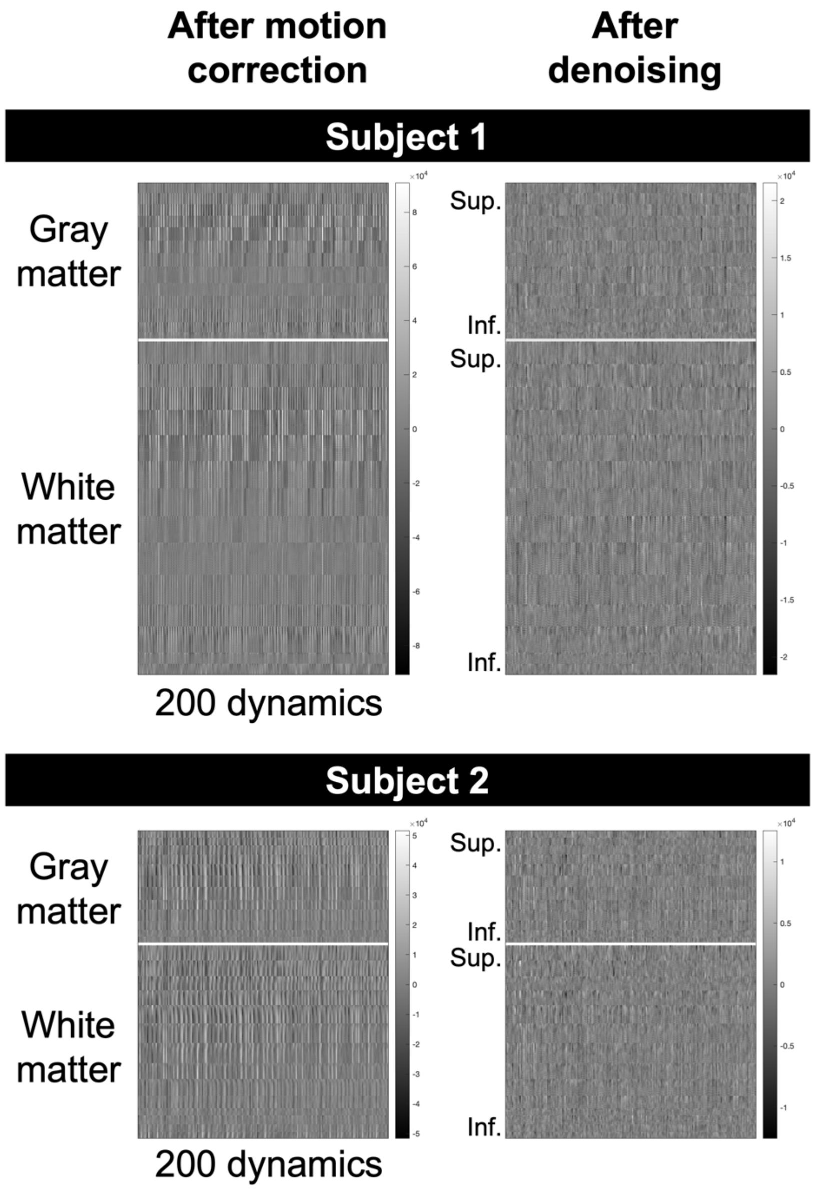

Supplemental Figure S7. Carpet plots are shown for two example subjects after motion correction and after nuisance removal (gray (GM) and white matter (WM) voxels from all 14 slices, shown in superior to inferior slice order). Note the size of the plots differs between subjects, as datasets have a different number of voxels per slice, due to anatomy and the number of available GM voxels in the lowest portion of the cord. The motion-corrected data show fluctuations of large amplitude, corresponding largely to breathing and which can be visually appreciated in the raw data. Those fluctuations are largely removed for both GM and WM voxels after the denoising step.
